# Intensive care unit admission in chronic obstructive pulmonary disease: patient information and the physician’s decision-making process

**DOI:** 10.1186/cc13906

**Published:** 2014-06-04

**Authors:** Matthieu Schmidt, Alexandre Demoule, Emmanuelle Deslandes-Boutmy, Marine Chaize, Sandra de Miranda, Nicolas Bèle, Nicolas Roche, Elie Azoulay, Thomas Similowski

**Affiliations:** 1 Sorbonne Universités, UPMC Univ Paris 06, UMR_S 1158 ‘Neurophysiologie Respiratoire Expérimentale et Clinique’, 47-83 boulevard de l'Hôpital, 75013 Paris, France; 2 INSERM, UMR_S 1158 ‘Neurophysiologie Respiratoire Expérimentale et Clinique’, 47-83 boulevard de l'Hôpital, 75013 Paris, France; 3 AP-HP, Groupe Hospitalier Pitié-Salpêtrière Charles Foix, Service de Pneumologie et Réanimation Médicale (Département ‘R3S’), 47 boulevard de l'Hôpital, F-75013 Paris, France; 4 Sorbonne Universités, UPMC Univ Paris 06, UMR_S 974, 47-83 boulevard de l'Hôpital, 75013 Paris, France; 5 INSERM, UMR_S 974, F-75005 Paris, France; 6 Hôpitaux de Paris, Hôpital Saint Louis, Service de Biostatistique, 1 avenue Claude Vellefaux, 75010 Paris, France; 7 Hôpitaux de Paris, Hôpital Saint Louis, Service de Réanimation Médicale, 1 avenue Claude Vellefaux, 75010 Paris, France; 8 Hôpitaux de Paris, Hôpital Cochin – Site Val de Grâce, Service de Pneumologie et Soins Intensifs Respiratoires, Université Paris Descartes, 27 Rue du Faubourg Saint-Jacques, F-75014 Paris, France

## Abstract

**Introduction:**

ICU admission is required in more than 25% of patients with chronic obstructive pulmonary disease (COPD) at some time during the course of the disease. However, only limited information is available on how physicians communicate with COPD patients about ICU admission.

**Methods:**

COPD patients and relatives from 19 French ICUs were interviewed at ICU discharge about their knowledge of COPD. French pulmonologists self-reported their practices for informing and discussing intensive care treatment preferences with COPD patients. Finally, pulmonologists and ICU physicians reported barriers and facilitators for transfer of COPD patients to the ICU and to propose invasive mechanical ventilation.

**Results:**

Self-report questionnaires were filled in by 126 COPD patients and 102 relatives, and 173 pulmonologists and 135 ICU physicians were interviewed. For 41% (n = 39) of patients and 54% (n = 51) of relatives, ICU admission had never been expected prior to admission. One half of patients were not routinely informed by their pulmonologist about possible ICU admission at some time during the course of COPD. Moreover, treatment options (that is, non-invasive ventilation, intubation and mechanical ventilation or tracheotomy) were not explained to COPD patients during regular pulmonologist visits. Pulmonologists and ICU physician have different perceptions of the decision-making process pertaining to ICU admission and intubation.

**Conclusions:**

The information provided by pulmonologists to patients and families concerning the prognosis of COPD, the risks of ICU admission and specific care could be improved in order to deliver ICU care in accordance with the patient’s personal values and preferences. Given the discrepancies in the decision-making process between pulmonologists and intensivists, a more collaborative approach should probably be discussed.

## Introduction

Chronic obstructive pulmonary disease (COPD) is an increasingly common cause of death [1]. At severe stages of the disease, episodes of acute respiratory failure often require intensive care unit (ICU) admission [2]. Although the corresponding acute mortality is relatively low [3] and lower than that of other diseases [4], outcomes after an exacerbation are poor [3,5,6]. Disease severity, comorbidities, and impairment of activities of daily living are salient prognostic factors [3,5,6]. Of note, intubation and invasive ventilation during an episode of exacerbation are associated with longer durations of stay and increased in-hospital and post-hospital mortality rates [7]. In this context, the American Thoracic Society/European Respiratory Society task force on COPD diagnosis and management [8] has recommended that ‘Healthcare providers should assist patients during stable periods of health to think about their advance care planning by initiating discussions about end-of-life care’ and stated that ‘these discussions should prepare patients with advanced COPD for a life-threatening exacerbation of their chronic disease …’ while ‘…providing information on probable outcomes and the existence of palliative interventions…’

Despite more stays in ICU and more resource-intensive care than patients with cancer [9], COPD patients are not always well informed about their disease in general and about the risk of ICU admission in particular. They are also poorly informed about what an ICU stay entails. Semi-structured interviews conducted in 21 patients with advanced COPD revealed that many of them were unaware of the progressive nature of the condition and few were aware that they could die from their disease [10]. Conversations about ICU care with COPD patients and their relatives during or after an acute episode are frequently conducted by intensivists rather than attending pulmonologists, in a context in which ICU stressors and post-traumatic disorders [11,12] can interfere with decisions, preferences and values. In addition, COPD patients are particularly prone to psychiatric disorders, with a high prevalence of anxiety [13,14]. As previously implemented in cancer patients, advance care planning could therefore improve the patient’s quality of life without inducing higher rates of major depressive disorder [15].

The first objective of this study was to provide a description of the information provided by pulmonologists to their COPD patients at regular follow-up visits and of the information received by COPD patients and their relative about COPD-related ICU stays. Because decision-making processes are bound to influence the information given to patients, we aimed to describe how pulmonologists based their decisions for ICU admission and intubation in comparison with intensivists.

## Material and methods

This study is an ancillary part of a previously published study [12] conducted in 19 French ICUs over an 18-month period after approval by the appropriate Institutional Review Board (*Comité de protection des personnes Ile de France 6*, La Pitié Salpêtrière, Paris, France). Completing the questionnaire was taken as evidence of consent to study participation. Data presented here concerning patients, relatives and pulmonologists have not been previously reported in a published article.

### Patient data

The previously published study [12] provided a detailed description of data collection and quality control procedures. Briefly, patients with COPD admitted to the 19 participating ICUs were prospectively screened and were included when they spent more than 24 hours in the ICU for COPD exacerbation. Exclusion criteria included ICU death, cognitive dysfunction, language barriers or severe psychiatric disorders. The patient’s relatives (one relative per patient) were included when they understood French. Collected variables included demographic characteristics, illness severity on admission, baseline psychological status and evaluation of anxiety or depression at ICU discharge [12]. At ICU discharge, patients and relatives filled in a questionnaire describing the information they had received about COPD before the acute episode and the terms used by their physicians (closed-ended questions).

### Physician data

A postal-based survey was administered to 200 pulmonologists randomly selected from the French language society of respiratory medicine (‘*Société de Pneumologie de Langue Française*’) database that comprises about 2,000 names. The survey was based on a case report and was designed to describe the physician’s practical approach to a 50-year-old 50 pack-year COPD patient with a forced expiratory volume in 1 second (FEV1) of 30% predicted. Questions pertained to general information about the disease, use of oxygen, and use of non-invasive and invasive mechanical ventilation. A 15-item questionnaire on COPD severity and outcomes was used in which each item was explored by a four-level Likert-type scale (‘never’ , ‘sometimes’ , ‘frequently’ and ‘always’). The questionnaire was returned by 173 of the 200 physicians surveyed (138 with no missing data).

Factors influencing decisions to refrain from proposing an ICU admission and invasive mechanical ventilation were also investigated by means of a 16-item questionnaire, in which each item was scored from 0 to 10 (with ‘0’ corresponding to ‘no impact on the decision’ and ‘10’ corresponding to ‘major impact on the decision’). This part of the questionnaire was also administered to 175 ICU physicians derived from the ‘Famirea’ database (135 responses, 119 with no missing data) [11].

### Statistical analysis

Statistical analysis was performed with SAS 9.1 software package (SAS institute Inc, Cary, NC, USA). The results are reported as median and interquartile range or as numbers and percentages. The baseline characteristics of the surveyed physicians - pulmonologists vs. intensivists - were compared using a chi-square test for categorical variables and a non-parametric Mann-Whitney test for continuous variables. The answers of the pulmonologists and intensivists to the 16-item questionnaire exploring factors influencing ICU admission and intubation decisions were studied according to principal component analysis [16,17], using Kaiser’s criterion to choose the number of components included [18]. Principal component analysis was based on questionnaires with no missing data (138 pulmonologists, 119 intensivists). This approach aims at reducing the complexity of a data set to lower dimensions by transforming the original coordinate system describing the data into a new set of coordinates (called ‘principal components’) with a common origin and perpendicular directions. The first principal component is a vector that points in the direction of highest variance. The second principal component points in the direction of the second highest variance, and so on. Relationships between variables are derived from the analysis of cell frequencies within a two-way contingency table. The relationships between one category of respondents (intensivists or pulmonologists) and their responses to the questionnaires determined a point of which the coordinates depend of the direction and strength of the association. The weights given to these items are higher when they have a positive Y-coordinate. Thus, in our study, each item in each category of responders was labelled as either a ‘high weight’ (positive Y-coordinate) or a ‘low weight’ item (negative Y-coordinate). In addition, we provide the Euclidean distance between the factors coordinates of a given component in the ‘intensivists’ data set and in the ‘pulmonologists’ data set. The higher the Euclidean distance, the larger the discrepancy between the item’s weight in the ‘intensivists’ data set and in the ‘pulmonologists’ data set.

Differences were considered significant when the probability *P* of a type I error was less than 5%.

## Results

### Characteristics of patients, relatives and physicians surveyed

**Figure 1:**
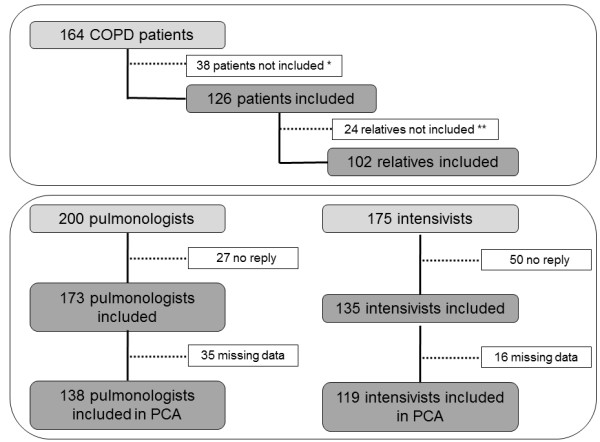
**Study flow chart.** *Reasons for non-inclusion of patients: death in ICU (n = 27), mental incompetence (n = 7), unexpected ICU discharge (n = 3) and refusal (n = 1); **among the 126 included COPD patients, 14 lived alone and had no relatives; among the 112 relatives potentially available to participate, seven could not speak French, and three refused to participate. COPD*,* chronic obstructive pulmonary disease; ICU, intensive care unit; PCA, principal component analysis.

One hundred and twenty six of the 164 patients with COPD admitted during the study period were included in the study (see flow chart on Figure 1). One hundred and two relatives were also included (Figure 1, Table 1). Figure 1 and Table 2 indicate the characteristics of the 173 pulmonologists and 135 intensivists. Most pulmonologists (84%) had gained experience in intensive care during their residency, while 41% of intensivists reported some training in pulmonology.

**Table 1:** Characteristics of patients and relatives

**Patients (n = 126)**	**Median (25th-75th) or n (%)**
Age, years	67 (57-75)
Male	79 (62)
WHO performance scale - stage 3 or 4^£^	71 (57)
Body mass index, kg.m^-2^	26 (22-32)
SAPS II	30 (23-40)
Symptoms of anxiety at baseline	31 (24)
Symptoms of depression at baseline	26 (20)
Time since COPD diagnosis, months	36 (9-240)
COPD management	
Regular general practitioner visits	110 (87)
Regular pulmonologist visits	76 (60)
First hospitalization for COPD exacerbation	35 (28)
First ICU admission	58 (46)
Had prior knowledge of respiratory disease	107 (84)
**Relatives (n = 102)**	
Tie with the patient	
Spouse	55 (54)
Children	10 (10)
Brother/sister	3 (3)
Father/mother	5 (5)
Other family tie	17 (17)
Friends	12 (11)
Only caregivers at home	38 (37)
Had prior knowledge of patient’s respiratory disease	87 (87)
Since, months	60 (24-120)

^£^WHO performance scale: World Health Organization performance scale to describe how well patients are. This score ranged from 0 to 5, is also called performance status [19]. COPD, chronic obstructive pulmonary disease; ICU, intensive care unit; SAPS II, Simplified Acute Physiology Score II.

**Table 2:** Characteristics of pulmonologists and intensivists

	**Pulmonologists (n = 173)**	**Intensivists (n = 135)**	***P***
Age (years)	50 (44-56)	35 (30-42)	<0.001
Male (n)	128 (77)	90 (67)	0.03
Practice			<0.001
university hospital	44 (25)	89 (66)	
general hospital	62 (36)	46 (34)	
private practice	67 (39)	0 (0)	
Past experience in pulmonology			<0.0001
None	0 (0)	80 (59)	
≤1 year	0 (0)	25 (18)	
1- 2 years	2 (1)	13 (10)	
>2 years	167 (99)	17 (13)	
Past experience in intensive care			<0.001
None	28 (16)	0 (0)	
≤1 year	76 (44)	24 (18)	
≤2 years	17 (10)	13 (10)	
>2 years	52 (30)	98 (72)	

### Communication between patients and pulmonologists

**Figure 2:**
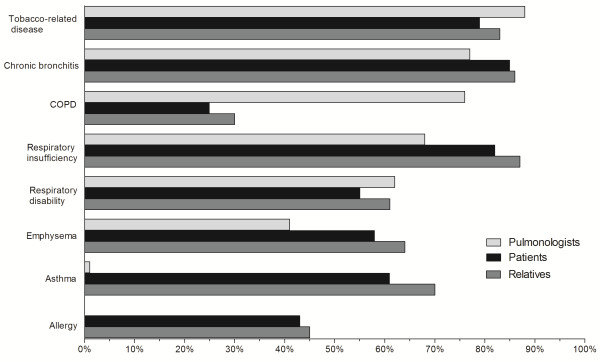
**Terminology used by patients, relatives and pulmonologists to discuss the disease.** The results are expressed as percentages of responses. COPD, chronic obstructive pulmonary disease.

Communication between COPD patients and pulmonologist was perceived as satisfactory by patients who attributed a score of 9/10 (IQR, 7 to 10) and by their relatives who attributed a score of 8/10 (5 to 9). Figure 2 compares the terms used by patients, their relatives and pulmonologists to discuss the disease. Patients and their relatives often reported use of the terms ‘emphysema’ and ‘respiratory insufficiency’ by their physician, but very rarely the use of ‘COPD’. Of note, ‘asthma’ and ‘allergy’ were used by more than 50% of patients and their relatives, although these terms were never used by pulmonologists. About 80% of physicians, patients and relatives used the expression ‘tobacco-related disease’.

**Figure 3:**
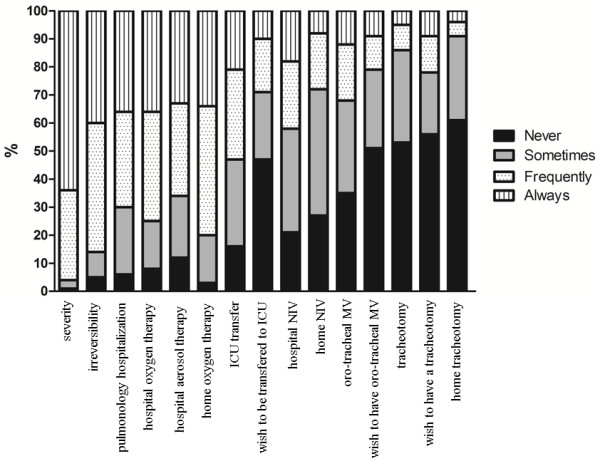
**COPD-related issues discussed by the surveyed pulmonologists with their patients.** COPD, chronic obstructive pulmonary disease; ICU, intensive care unit; MV, mechanical ventilation; NIV, non-invasive ventilation.

For 31% of patients (n = 39) surveyed, ICU admission had never been expected. Only 56% reported having discussed this possibility with their general practitioner and/or pulmonologist. Only 54% of relatives (n = 51) surveyed were aware that the patient could possibly require ICU admission.Figure 3 describes the frequency with which the surveyed pulmonologists reported informing their COPD patients about the 15 items of the severity and outcomes questionnaire. The severity and irreversibility of the disease was mentioned almost systematically. The risk of an exacerbation requiring a hospital stay in a respiratory medicine ward was mentioned ‘always’ or ‘frequently’ in more than two-thirds of cases. In contrast, the possibility of an ICU admission was mentioned ‘always’ or ‘frequently’ in about one-third of cases, and ‘never’ in 50% of cases. The possibility of intubation was never or only sometimes mentioned in 70% of cases. The patient’s preferences concerning ICU admission, intubation and tracheotomy were never or only sometimes mentioned in >70% of cases.

### Factors contributing to the physicians’ attitude toward ICU admission and intubation

Tables 3 and 4 shows the results of principal component analysis.

**Table 3:** Factors negatively influencing decisions of non-admission to the ICU among pulmonologists and intensivists (16-item questionnaire)

	**High weight for pulmonologists**	**High weight for intensivists**	**Euclidean distance**
Respiratory nurse’s opinion	+	-	2.804
Other pulmonologist/intensivist’s opinion	+	-	2.686
General practitioner’s opinion	+	-	2.505
Home non-invasive ventilation	-	+	2.447
Family’s opinion	+	-	2.444
Home oxygen	-	+	2.256
Patient’s opinion	+	-	2.187
No family	+	-	1.924
Forced expiratory volume in 1 second <30% predicted	-	+	1.757
Number of hospitalizations in last year	-	+	1.381
Number of hospitalizations with mechanical Ventilation in last year	-	+	1.293
Depression	+	+	0.908
Smoking cessation	+	+	0.792
Heart failure	+	+	0.570
Age	-	-	0.385
Physician’s perception of the patient’s quality of life	-	-	0.330

These data were obtained by principal component analysis. Large Euclidean distances indicate substantial differences between pulmonologists’ and intensivists’ responses, while short distances indicate similarity. The mark ‘+’ indicates a positive Y-coordinate in the principal component analysis which means an important weight given to this item by the ‘pulmonologists’ data set or the ‘intensivists’ data set. Conversely, the mark ‘-’ displays a negative Y-coordinate which indicates a low item’s weight.

**Table 4:** Factors negatively influencing decisions of non-intubation among pulmonologists and intensivists (16-item questionnaire)

	**High weight for pulmonologists**	**High weight for intensivists**	**Euclidean distance**
General practitioner’s opinion	-	+	1.898
Smoking cessation	+	-	1.639
Physician’s perception of the patient’s quality of life	+	-	1.587
No family	-	+	1.547
Respiratory nurse’s opinion	+	+	1.237
Heart failure	-	+	1.222
Depression	+	+	0.916
Number of hospitalizations in last year	-	-	0.833
Age	+	+	0.735
Home oxygen	-	-	0.702
Family’s opinion	+	+	0.594
Forced expiratory volume in 1 second < 30%	-	-	0.566
Predicted number of hospitalizations with mechanical ventilation in last year	-	-	0.496
Other pulmonologist/intensivist’s opinion	+	+	0.477
Home non-invasive ventilation	-	-	0.475
Patient’s opinion	+	+	0.331

These data were obtained by principal component analysis. Large Euclidean distances indicate substantial differences between pulmonologists’ and intensivists’ responses, while short distances indicate similarity. The mark ‘+’ indicates a positive Y-coordinate in the principal component analysis, which means an important weight given to this item by the ‘pulmonologists’ data set or the ‘intensivists’ data set. Conversely, the mark ‘-’ displays a negative Y-coordinate which indicates a low item’s weight.

### ICU admission

The largest Euclidean distances between the answers of the pneumologists and that of the intensivists were noted for the items related to personal views (that is ‘general practitioner’s opinion’; ‘patient’s opinion’; ‘family’s opinion’; ‘respiratory nurse’s opinion’; ‘another pulmonologist’s opinion’). The weights attributed to these items by pulmonologists were higher than those attributed to the same items by intensivists (Table 3). Similar differences were also observed for ‘no family’. Pulmonologists’ and intensivists’ responses were also in opposite direction for items related to medical status (that is ‘FEV1 < 30% predicted’; ‘home oxygen’; ‘number of hospitalizations during the last year’; ‘number of hospitalizations with mechanical ventilation during the last year’). Intensivists attributed a higher weight to these items than pulmonologists (Table 3).

### Intubation

The differences between pulmonologists and intensivists regarding intubation were less marked than those concerning ICU admission. The ‘opinion-related’ items and ‘age’ were given similar - and high - weights by both categories of physicians (Table 4). Pulmonologists’ and intensivists’ responses displayed discrepancies concerning ‘physician’s personal perception of patient’s quality of life’ and ‘smoking cessation’ , as pulmonologists attributed higher weight to these items than intensivists, while an opposite trend was observed for the ‘heart failure’ item.

## Discussion

This study highlights some of the barriers to patient-physician dialogue concerning COPD in general, and severe acute episodes of the disease in particular, despite the fact that patients and pulmonologists both rated the quality of their reciprocal communication as being very high. This study also shows that pulmonologists and intensivists have different perceptions of the decision-making process pertaining to these episodes, providing insight to future approaches designed to improve information of COPD patients about their disease.

The vocabulary used by patients, relatives, and pulmonologists to describe the disease illustrates the communication difficulties related to COPD. The three categories of respondents used ‘chronic bronchitis’ , ‘emphysema’ and ‘respiratory insufficiency’ with comparable frequencies, which is a positive result. However, COPD patients and their relatives fairly frequently used the terms ‘asthma’ and ‘allergy’ , which indicates a certain degree of confusion, suggesting that pulmonologists were not their unique source of information. These observations are consistent with previously published studies indicating that COPD patients have a poor knowledge of their disease [20-22]. In particular, COPD patients also have a poor understanding of the term ‘exacerbation’ [23].

The findings of this study also indicate that the risk of severe COPD exacerbations (those requiring ICU admission and mechanical ventilatory assistance) is a difficult subject to discuss with patients and relatives. Fifty percent of the surveyed pulmonologists reported that they never or only ‘sometimes’ informed their patients corresponding to this clinical setting that they were at risk of ICU admission in the event of a severe exacerbation. These rates increased to 60% concerning non-invasive ventilation, 70% concerning intubation, and 90% concerning tracheotomy. Of note, the surveyed pulmonologists were not asked to go beyond the case report and describe how disease severity influenced the information they gave to their patients. In line with the above findings, 31% of patients (n = 39) and 54% of relatives (n = 51) reported that they had not previously expected ICU admission. Bearing in mind that 54% (n = 68) of patients in this study had been previously admitted to an ICU and were therefore unlikely to be unaware of this possibility, this figure of 31% appears to be extremely high. In contrast, it is likely that almost all COPD patients in the study with no previous ICU stay were unprepared for this possibility.

As critically ill patients are usually unable to make decisions and as nearly one half of surrogates do not understand the concept of surrogate decision-making [24,25], it would be particularly useful to discuss the COPD patient’s preferences and advanced care planning. However, only a small proportion of the surveyed pulmonologists reported discussing the patient’s preferences in relation to ICU admission, intubation, and tracheotomy (Figure 3), which is consistent with recent literature on this issue [10,26-29]. Nevertheless, many COPD patients express the desire to discuss these issues [29] and pulmonologists in the present study attributed high weights to items related to ‘the patient’s or family’s personal views’ during decision-making processes concerning ICU admission and intubation. There are a number of barriers to advanced ICU care planning for COPD patients. The main barriers acknowledged by pulmonologists during end-of-life discussions were: ‘too little time during the appointment’, ‘a desire to preserve the patient’s hope’ , ‘a feeling that the patient was not ready to talk about the care she/he wants’ [27]. Few studies have been devoted to communication barriers identified by pulmonologists concerning the risk of ICU admission and the resulting prognosis, despite the recent recommendations in the Global Initiative for Chronic Obstructive Lung Disease (GOLD) report [30]. Some of these barriers may be lack of time, lack of intensive care experience, difficulties talking about another speciality or discomfort with giving bad news. Moreover, terms are confusing (Figure 2) and patients may often be reluctant to ask questions. Thus pulmonologists may feel they have communicated information while the patient may feel they received little. Several simple measures, mostly based on improving communication strategies, could be implemented. For instance, including non-pulmonologists in discussions [31], informative handouts to educate families about critical illness and intensive therapies [32], as well as proactive and effective communication strategies [24,33] could possibly overcome these pitfalls. Preparing COPD patients for the risk of severe exacerbations, as well as defining their preferences in relation to ICU admission, intubation and tracheotomy could have a beneficial impact on their perceived overall quality and satisfaction with care [34-36] and could also decrease the high psychological burden of ICU on COPD patients and their relatives [12,15].

Prognostic factors of COPD have been clearly identified [3,5,6,37]. Informing COPD patients about ICU admission and the subsequent methods of care necessarily implies taking these prognostic factors into account. However, the results of this study indicate that the pulmonologists surveyed, compared to intensivists, attributed more weight to items related to ‘personal views’ (of the patient, family, other healthcare practitioners) and less weight to more objective elements, including well-documented prognostic factors (such as low FEV1, home oxygen, and so on). Between-specialist heterogeneities in the care of COPD patients are well known [38-40] and could help to explain the results of this study. It can be postulated that it is more difficult to choose when and how to address end-of-life related issues such as ICU admission with patients when decision-making criteria are subjective rather than based on objective criteria. It is noteworthy that the nature of the information given to patients depends on the clinician’s evaluation of the prognosis: in the field of COPD, there is evidence that an unwarranted prognostic pessimism can be a source of under-treatment [41]. Pulmonologist-intensivist differences were less marked in relation to the intubation decision-making process than in relation to the ICU admission decision-making process. These results highlight the need for closer collaboration between pulmonologists and intensivists. Multidisciplinary meetings, systematic experience of intensive care during pulmonology residencies and post-ICU consultation by intensivists, should be discussed.

This study presents several limitations. The pulmonologists surveyed were not the healthcare providers of the patients interviewed. An in-depth evaluation of COPD knowledge was not conducted, but the assessment was restricted to the terms used to explain COPD in a specific population with severe COPD following an ICU stay. In addition, our study focused on ICU survivors. Thus, we do not know about the information given to the non-survivors of critical illness. Since they were more severely ill, we cannot rule out they might have received more information in advance. The way in which pulmonologists and intensivists stratify their decision-making process according to disease severity and the decision-making process concerning major ICU issues in COPD, such as non-invasive ventilation and tracheostomy that may also have been relevant were also not investigated.

## Conclusions

Nevertheless, our observations confirm that further effort is required to enable COPD patients to play a fully informed role in end-of-life decisions and more specifically in decisions concerning ICU admission and intubation, while keeping encouragement and support to maintain life as productive as possible. The nature and timing of this communication should probably be more clearly defined, and should take into account the known objective prognostic factors. Given the discrepancies in the decision-making process between pulmonologists and intensivists, a more collaborative approach should probably be discussed. Further interventional studies are now warranted.

## Key messages

• Patients with COPD have a poor knowledge of their disease.

• Only a small proportion of pulmonologists report discussing the patient’s preferences in relation to ICU admission, intubation, and tracheotomy.

• Pulmonologists and ICU physicians have different perceptions of the decision-making process pertaining to ICU admission and intubation.

• Further efforts are required to enable COPD patients to play a fully informed role in end-of-life decisions and more specifically in decisions concerning ICU admission and intubation.

## Abbreviations

COPD: chronic obstructive pulmonary disease; FEV1: forced expiratory volume in 1 second; ICU: intensive care unit.

## Competing interests

The authors declare that they have no competing interests.

## Authors’ contributions

MS is the guarantor of the manuscript and takes responsibility for the integrity of the work as a whole, from inception to published article. MC contributed to data collection, data analysis, and the revision of the manuscript. SDM contributed to data collection and the revision of the manuscript. NB contributed to data collection and the revision of the manuscript. NR contributed to data collection and the revision of the manuscript. EA contributed to data collection, data analysis and the revision of the manuscript. MS contributed to data analysis, statistical analysis, and the writing and revision of the manuscript. AD contributed to data analysis, statistical analysis and the revision of the manuscript. EBD contributed to data analysis, statistical analysis, and the writing and revision of the manuscript. TS contributed to data analysis, and the writing and revision of the manuscript. All authors read and approved the final manuscript.

## Authors’ information

MS is an ICU physician. AD is a professor of intensive care.EBD is a statistician. MC is an ICU fellow. SDM is a pulmonologist. NB is an ICU physician. NR is a professor of pulmonology. EA is a professor of intensive care. TS is a professor of pulmonology. He is the chair of the pulmonology department and medical ICU in the Pitie Salpetriere hospital in Paris.
